# Bacteriology Run to Extremes

**Published:** 1891-01

**Authors:** 


### Bacteriology Run to Extremes.

The *Medical Record*, August 30, 1890, says editorially: Is it not about time to halt a while in our restless search after the microscopical accompaniments of certain diseases, and pause to consider what we are accomplishing, and whether there is not some danger of becoming men of one idea, narrow to the extreme in our conception of what disease actually is, and of how we ought to combat its ravages. It may be that we are not different from other men, yet it would seem as though medical men might be justly called the Athenians among men of science, in that they "employ themselves in nothing else, but either in telling or in hearing some new thing," and when they have found that they let every thing else go and cling to that alone. It is thus with the study of bacteriology. It has been ascertined almost to a certainty that the tubercular process, for example, is associated with a certain form of microscopic growth which is not found as an accompaniment of other diseases. It is therefore assumed, correctly no doubt, that this micro-parasite is the cause, either directly or indirectly, of tuberculosis. Other microbes have been found in relation with other diseases, and it has also been assumed that a causal nexus exists. This is well enough, it is a plausible theory, and one that offers great hope of advance in the therapy of certain diseases, and if it does not blind us to the actual necessities of treatment and lead us to neglect the plain indications while running after some specific microbicide, there is no objection to its further study and amplification. But here is where the tendency of mankind, in general, and seemingly of the medical profession, in particular, to ride a hobby to death betrays itself. Some are not satisfied in accepting what has already been proven, but they must assume what remains to be, and which probably never will be proven. They look for the bacillus in every departure of the human organism from health, and they run a serious risk of making themselves, as well as the science which they study ridiculous. Not long ago it was gravely announced in an Italian journal that some one had discovered the bacillus of old age. Of course that was intended as a joke, but so ready at the present day are some minds to see the infinitesimal in pathology that not all could see the joke, and several medical papers reproduced the item containing the announcement of the great discovery in all seriousness, never for a moment seeming to see in it any thing but the most natural discovery, and one that would explain many of the lesions found in those of advancing years. But there are other things put forward in good faith which are almost as ridiculous. Not long since a Russian investigator examined the water obtained from melted hailstones and found it to contain several varieties of bacteria. There was nothing very remarkable in this discovery, for it would have been strange had no bacteria been found, but what was remarkable was the conclusion which the man drew. He thought it very probable, since rain, snow, and hail had all been found to contain micro-organisms, that there was a specific, and hitherto undescribed, disease to which only those were liable who had been exposed to a storm and had been wet to the skin by water containing these peculiar bacilli. It is not the search after microbes that is absurd, but the conclusions which many run to without first establishing their premises. We are not yet in position to assert that there can be no disease without its specific micro-organism, and those who do so maintain are in danger of making their science a subject of ridicule to men whose range of vision extends beyond the microscope field. They also interfere with therapeutic progress by turning men's minds away from the phenomena of diseased action and hunting after an invisible foe, which may after all be found to have less power for evil than it is now credited with.

A CEMENT for slides holding preparations in glycerine is prepared by Dr. S. Apathy (*Zeitsch. Wiss. Mikr.*) by melting together equal parts of Canada balsam and paraffin (60° melting point), and continuing the heat over a moderate flame until terebinthinous vapors are no longer perceptible, and the mixture has assumed a golden yellow hue. The mass, hard when cold, is applied by first melting again.

